# Editorial: Microbial Regulation of Translation

**DOI:** 10.3389/fgene.2020.616946

**Published:** 2020-11-23

**Authors:** Assaf Katz, Sebastian A. Leidel, Michael Ibba

**Affiliations:** ^1^Programa de Biología Celular y Molecular, Instituto de Ciencias Biomèdicas, Facultad de Medicina, Universidad de Chile, Santiago, Chile; ^2^Research Group for RNA Biochemistry, Department of Chemistry and Biochemistry, University of Bern, Bern, Switzerland; ^3^Schmid College of Science and Technology, Chapman University, Orange, CA, United States

**Keywords:** translation, ribosome, tRNA, regulation, microorganisms

Since the description of the operon model by Jacob and Monod during the late 1950s and early 1960s (Ullmann, 2010), the concept that the reading of genetic information must be a regulated process has been central to our understanding of biology. This is particularly true for microbes, which can adapt to an incredible variety of environments. Based on the research performed since the description of the operon, we have gained a deep understanding of the diverse strategies used by microbes to modulate the transcription of genetic information from DNA to RNA. In contrast, the mechanisms that regulate the translation of messenger RNAs into proteins has received less attention. The technical developments of the last decade now allow us to obtain detailed information on RNA folding (Rouskin et al., 2014; Aw et al., 2016) and modification (Linder et al., 2015; Lorenz et al., 2020) and the speed of translation (Subramaniam et al., 2013; Ingolia, 2014; Dai et al., 2016). This, in turn, allows us to scrutinize the functionality of translation components *in vivo*, providing unprecedented opportunities to study translation regulation. In this special issue of Frontiers in Genetics, “Microbial Regulation of Translation,” we have assembled a series of articles that use diverse experimental approaches to study the regulation of translation in microbes.

Some of the papers in this issue focus on alterations of translation derived from changes in ribosome function and abundance. For instance, Pletnev et al. studied the physiological and molecular effects of mutating all genes known to methylate nucleotides of rRNA in *Escherichia coli*. While the mutation of some genes strongly impacts bacterial replication, others only lead to minor effects. Interestingly, with the exception of the *rsfM* mutation, most mutants exhibit defects in translation when the system is challenged by overexpression of exogenous genes, although some of these strains show only small effects under “normal” conditions. The article by Yoshida et al. is also related to changes in ribosome availability in *E. coli*. Nevertheless, this work focuses on the natural regulation of ribosome availability through hibernation and how this is coordinated with the abundance of RNA polymerase and its diverse sigma factors.

Other articles in this issue study the regulation of the initiation and elongation steps of translation. For instance, one article (Radío et al.) shows how ribosome profiling can be used to study the regulation of translation initiation by uORFs in *Trypanosoma cruzi*, a mechanism that accounts for regulation of almost 10% of the genes. Other articles instead discuss regulation of translation elongation. Leiva et al. shows how inactivation of tRNA^Gly^ under oxidative stress may regulate translation elongation in *E. coli*, thereby changing protein synthesis only under specific environmental conditions. Using a different bacterial model, *Mycobacterium smegmatis*, Barth and Woychik analyze the role of the toxin-antitoxin system MazEF-ms in the regulation of translation. They found that MazEF-ms cleaves tRNA^Lys^, thereby decreasing the speed of translation elongation at AAA Lys codons. Thus, when this toxin is activated, expression of AAA rich genes is decreased while expression of AAA poor genes is increased.

In addition to the paper by Barth and Woychik, two reviews in this issue analyze the effects of diverse toxins on the translation machinery. Jurenas and van Melderen focus on the ability of many type II toxin-antitoxin systems to interfere with translation. These systems are composed of a toxin that may inhibit central processes of a cell and its antitoxin, a labile protein that inhibits the activity of the toxin. The authors discuss how most of these toxins' targets are components of the translation apparatus, including mRNA, tRNA, ribosomes, and translation factors. They further propose that the huge variability of these systems derives from low selective pressure on bacteria to maintain them, and the high selective pressure on the toxin-antitoxin systems to diverge from similar systems allowing lateral transfer to organisms carrying similar toxin-antitoxins. Thus, they propose these are “selfish genes” that usually give little advantage to bacteria. The second review about toxins uses a very different approach. The text written by Travin et al. is focused on ribosomally synthesized and post-translationally modified peptides (RiPPs). Similar to type II toxin-antitoxin systems, many of the RiPPs that have been described target diverse components of the translation apparatus. Nevertheless, in contrast to type II toxin-antitoxin systems, these compounds are not targeted to inhibit the self-translation machinery, but that of competitor organisms. Travin et al. provide an in-depth description of several of these compounds from a structural and functional perspective and highlight strategies to screen for pathways that produce new varieties of these compounds and their potential pharmacological use.

Finally, two papers in this issue use an unbiased screening to identify patterns in regulation of gene expression. Gummesson et al. describe the use of spike-in normalized RNA sequencing, applying it to *E. coli* cells subjected to valine-induced isoleucine starvation. In addition to providing valuable biological data, they show how changes in the total RNA levels may interfere with typical normalization protocols used to analyze transcriptomic data. Finally, Zhao et al. describe the usage of combined proteomics and genomics to study the relation between the organization of genes in operons on their cellular concentrations. They find interesting correlations, particularly for operons coding genes from a single complex or metabolic pathway.

In total, the collection of papers included in this issue represents the enormous variety of approaches and findings of an area that has been invigorated by the developments of the last decade.

## Author Contributions

AK wrote the paper with contributions from SL and MI. All authors contributed to the article and approved the submitted version.

## Conflict of Interest

The authors declare that the research was conducted in the absence of any commercial or financial relationships that could be construed as a potential conflict of interest.
